# Targeting Mitochondrial Metabolic Dysfunction in Pulmonary Hypertension: Toward New Therapeutic Approaches?

**DOI:** 10.3390/ijms24119572

**Published:** 2023-05-31

**Authors:** Marianne Riou, Irina Enache, François Sauer, Anne-Laure Charles, Bernard Geny

**Affiliations:** 1 Translational Medicine Federation of Strasbourg (FMTS), CRBS, University of Strasbourg, Team 3072 “Mitochondria, Oxidative Stress and Muscle Protection”, 1 Rue Eugène Boeckel, CS 60026, CEDEX 67084 Strasbourg, Franceirina.enache@chru-strasbourg.fr (I.E.); francois.sauer@chru-strasbourg.fr (F.S.); anne.laure.charles@unistra.fr (A.-L.C.); 2 Physiology and Functional Exploration Unit, University Hospital of Strasbourg, 1 Place de l’Hôpital, CEDEX 67091 Strasbourg, France; 3 Cardiology Unit, University Hospital of Strasbourg, 1 Place de l’Hôpital, CEDEX 67091 Strasbourg, France

**Keywords:** pulmonary hypertension, pulmonary vascular remodeling, right ventricle, mitochondrial dysfunction, Warburg effect, metabolic abnormalities

## Abstract

Pulmonary arterial hypertension (PAH) is a rare disease characterized by pulmonary vascular remodeling leading to right heart failure and death. To date, despite the three therapeutic approaches targeting the three major endothelial dysfunction pathways based on the prostacyclin, nitric oxide/cyclic guanosine monophosphate, and endothelin pathways, PAH remains a serious disease. As such, new targets and therapeutic agents are needed. Mitochondrial metabolic dysfunction is one of the mechanisms involved in PAH pathogenesis in part through the induction of a Warburg metabolic state of enhanced glycolysis but also through the upregulation of glutaminolysis, tricarboxylic cycle and electron transport chain dysfunction, dysregulation of fatty acid oxidation or mitochondrial dynamics alterations. The aim of this review is to shed light on the main mitochondrial metabolic pathways involved in PAH and to provide an update on the resulting interesting potential therapeutic perspectives.

## 1. Introduction

Pre-capillary pulmonary hypertension (PH) is defined as increased mean pulmonary artery pressure (PAP) > 20 mmHg associated with pulmonary capillary wedge pressure (PCWP) ≤ 15 mmHg and pulmonary vascular resistance (PVR) > 2 Wood units at rest, measured by right heart catheterization [1]. Among the types of pre-capillary PH, pulmonary arterial hypertension (PAH) corresponds to group 1 of the classification of PH and is a rare disease with a complex pathophysiology involving endothelial dysfunction, the proliferation of smooth muscle cells (SMCs), genetic predisposition, autoimmunity, and inflammation leading to pulmonary vascular remodeling [2]. Ultimately, these vascular changes lead to right ventricle (RV) remodeling, heart failure, and death. PAH arises from various causes, including idiopathic or hereditary causes, connective tissue diseases or exposure to drug or toxins.

Despite significant progress in the understanding of pathogenesis in PAH, the disease is diagnosed at a late stage, which prevents the implementation of early treatment to slow its progression. Over the past 15 years, three therapeutic approaches based on vasodilators targeting the mediators of pulmonary endothelial dysfunction (prostacyclin, nitric oxide [NO]/cyclic guanosine monophosphate [GMP], and endothelin pathways) have been identified, leading to the marketing of about 10 drugs. Recent advances in the treatment of PAH have shown the value of combining these treatments in an intensive strategy depending on the severity of the disease [1]. Thus, a triple therapy will be initiated in the most severe patients. However, despite these therapeutic innovations, PAH remains a serious disease with a 5-year survival rate of 60%. Therefore, new targets and therapeutic agents are needed.

According to numerous published papers, mitochondrial dysfunction is a therapeutic target of increasing interest in PAH [3]. The mitochondrion is the energy powerhouse of all eukaryotic cells through oxidative phosphorylation (OXPHOS). It has its own deoxyribonucleic acid (DNA) and plays an essential role in intra- and intercellular communication, the regulation of apoptosis, calcium homeostasis, and various metabolic pathways. Thus, defects or the dysregulation of mitochondria lead to a disproportionate inflammatory response, immune dysregulation, and abnormal tissue remodeling. In PAH, mitochondria play an essential role in pulmonary vascular remodeling, and the metabolic switch from OXPHOS energy production to glycolysis in all pulmonary vascular cells and in the RV appears to be one of the major mechanisms involved in the disease. Furthermore, in the pulmonary vasculature, mitochondria are a physiological source of reactive oxygen species (ROS) and act as oxygen sensors involved in pulmonary hypoxic vasoconstriction. Based on these data, many interesting potential therapeutic pathways are currently under investigation.

The aim of this review is to shed light on the main mitochondrial metabolic pathways involved in PAH and to review the potential therapeutic perspectives arising from them. The dysregulation of calcium homeostasis and the mechanisms of ROS involvement in PAH will not be detailed in this review.

## 2. Mitochondrial Metabolic Dysfunction and PAH

**Figure 1:**
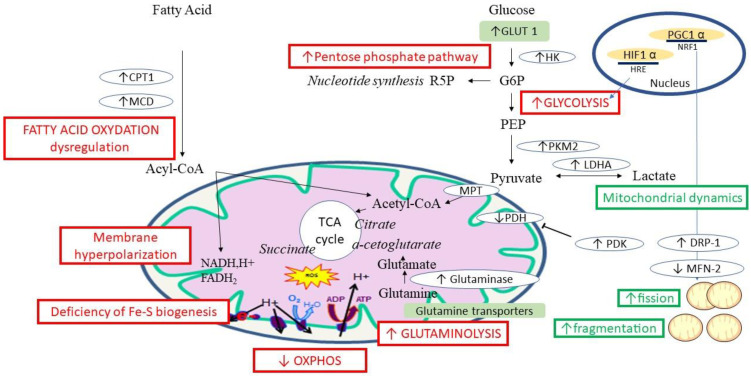
Main mitochondrial metabolic pathways involved in PAH. Acyl-CoA: acyl-coenzyme A; CPT-1: carnitine palmitoyltransferase 1; DRP-1: dynamin-related protein 1; Fe–S: iron-sulfur; GLUT 1: glucose transporter 1; G6P: glucose-6-phosphate; HIF: hypoxia inducible factor; HK: hexokinase; HRE: hypoxia response element; LDHA: lactate dehydrogenase; MCD: malonyl-CoA decarboxylase; MFN-2: mitofusin-1; MPT: mitochondrial pyruvate transporter; PDH: pyruvate dehydrogenase, PDK: pyruvate dehydrogenase kinase; PEP: phosphoenolpyruvate; PGC 1: Peroxisome proliferator-activated receptor-gamma coactivator; PKM2: pyruvate kinase isoform M2; TCA cycle: tricarboxylic acid cycle.

The major mitochondrial metabolic pathways involved in PAH are shown in Figure 1.

### 2.1. The Warburg Effect

Mitochondrial dysfunction and metabolic reprogramming are components of PAH pathogenesis, observed in both pulmonary arteries and RV [4,5]. In contrast to healthy cells that rely primarily on mitochondrial OXPHOS for energy production (i.e., adenosine 5′-triphosphate [ATP] production), pulmonary vascular cells in PAH (i.e., SMCs, endothelial cells [ECs], and fibroblasts) behave like cancer cells and use glycolysis, even in the presence of oxygen, for energy production [6,7,8]. Under normal conditions, glycolysis and glucose oxidation are coupled, and the end products of glucose are ATP and pyruvate. After pyruvate passes into the mitochondria via the mitochondrial pyruvate transporter (MPT), it is converted by pyruvate dehydrogenase (PDH) to acetyl coenzyme A (acetyl-CoA), which fuels the tricarboxylic acid cycle (TCA cycle, also known as the Krebs cycle).

In PAH, pyruvate is converted to lactate by lactate dehydrogenase (LDHA) due to PDH inhibition, resulting in less ATP production (N = 2 molecules) compared to the OXPHOS pathway (N = 38). This metabolic detour phenomenon was described by Otto Warburg in 1924 and is referred to as aerobic glycolysis or the “Warburg effect” [9]. The Warburg metabolism involves two mitochondrial failures: glucose metabolism and oxygen sensing. This effect promotes a cancer-like phenotype in the vascular cells of pulmonary arteries, with hyperproliferation and impaired apoptosis. The key enzymes in this metabolism shift are PDH and PDK 1 and 2 (pyruvate dehydrogenase kinase 1/2). In PAH, there is an upregulation of PDK, which phosphorylates and inhibits PDH, thereby reducing acetyl-CoA production. Pyruvate kinase (PK), the last key enzyme in the conversion of glycolysis (which catalyzes the transfer of phosphate from phosphoenolpyruvate to adenosine diphosphate (ADP) and synthesizes pyruvate), is also dysregulated in PAH. In addition, the downregulation of MPT decreases the mitochondrial amount of pyruvate.

The mechanisms leading to Warburg metabolism are not fully understood. A pseudo hypoxic state with normoxic activation of the subunit alpha of the hypoxia inducible factor (HIF-1 α) appears to be the primary factor involved. The role of HIF is easily understood in PH secondary to chronic hypoxia (group 3 of the classification) since HIF-1 α is directly activated under hypoxic conditions, but normoxic activation of HIF-1 α has also been described in PAH [10]. Mitochondrial ROS could also modulate HIF-1 α expression [11,12]. The upregulation and stabilization of HIF-1 α induce the expression of glycolysis proteins such as PDK, glucose transporter 1 (GLUT 1), hexokinase (HK) or LDHA. In addition, and although its role in the Warburg effect is unclear, HIF-2 α regulates some of the same genes of HIF-1 α, suggesting a potential role in the regulation of the glycolytic pathway [13]. However, the exact upstream and downstream mechanisms of HIF signaling are not fully understood. Targeting HIF directly would be an attractive therapeutic approach, but further investigations are needed.

In 2017, two additional regulators of the metabolic switch were described [14]. Caruso et al. and Zhang et al. demonstrated that glycolysis was stimulated by the upregulation of a ribonucleoprotein, called polypyrimidine tract-binding protein (PTBP1), which inhibits the PKM1 isoform and increases PKM2 [15,16]. An increase in the PKM2/PKM1 ratio leads to glycolysis in animal models of PAH and in the pulmonary vascular cells of PAH patients. Micro-RNA-124 (Mir124) is thought to be involved in this mechanism.

Most interestingly, the glycolytic switch in the PAH vasculature can be detected by (18)F-fluorodeoxyglucose positron emission tomography (FDG-PET) scans and correlates with disease severity [17,18]. Cardiomyocytes are also affected by this effect, resulting in RV hypertrophy, reduced contractility, and apoptosis [19].

Nevertheless, the properties of pulmonary vascular cells in PAH patients differ from those of cancer cells, which have the characteristic of proliferating at a distance. In PAH, the proliferative involvement is limited to the pulmonary vessels (as well as to the RV for some metabolic features). Moreover, no recurrence of PAH has been described after lung transplantation. It is therefore rather a pseudotumor phenotype of pulmonary vascular cells in PAH.

### 2.2. Upregulation of Glutaminolysis and TCA Cycle Dysfunction

Abnormalities in the pathway before OXPHOS may also facilitate the Warburg effect [20]. A global study of plasma metabolites in PAH indicated an accumulation of acylcarnitine, glutamate (precursors of molecules that enter the TCA cycle), and TCA cycle intermediates [21], suggesting a dysfunction of the TCA cycle or at least the inability to meet the demands of proliferating pulmonary vascular cells in PAH [22,23]. Furthermore, the hypothesis of dysfunctional energy metabolism is strengthened by the increased levels of citrate, succinate, and fatty acid metabolites demonstrated in the lung tissue of PAH patients [22]. Increased glutaminolysis appears to be another mechanism of the proliferation of pulmonary vascular cells in PAH [24]. Glutamine is the most abundant circulating amino acid in blood and muscle. It is essential for the synthesis of metabolites that maintain mitochondrial metabolism and the activation of cell signaling. Glutaminolysis is one of the pathways for restocking the carbon intermediates of the TCA cycle and contributes to the anaplerosis reaction. Increased glutaminolysis involves glutaminase 1 (GLS1) upregulation, which is involved in the deamination of glutamine to glutamate, and the uptake of glutamine by the PAH vasculature. These hypotheses are reenforced by the fact that PAH patients with mutations in the *BMPR2* gene show increased glutamate uptake in ECs [25]. Glutaminolysis is also upregulated in the RV, as demonstrated in animal models of monocrotaline-induced RV hypertrophy [26]. Thus, targeting abnormal glutamine metabolism appears to be a promising therapeutic pathway in PAH [27].

### 2.3. Dysregulation of Fatty Acid Oxidation (FAO)

Fatty acid metabolism corresponds to different pathways such as cellular fatty acid uptake and storage, fatty acid synthesis, fatty acid transport in mitochondria, and FAO. In cardiomyocytes, FAO generates 60 to 90% of ATP production, and the remaining 10 to 40% of energy is generated from glycolysis and glucose oxidation. The balance between these two processes is called the Randle cycle [28,29]. Fatty acids entering the mitochondria undergo β-oxidation during which they are converted to acetyl-CoA and enter the TCA cycle to be converted to citrate. In response to citrate production, phosphofructokinase is inhibited, and glucose-6-phosphate levels increase and inhibit hexokinase, inducing a reduction in pyruvate production. In addition, acetyl-CoA production due to FAO inhibits PDH. Similarly, the inhibition of FAO induces a metabolic shift toward glycolysis [30].

In PAH, fatty acid metabolism abnormalities are described in blood, RV, and pulmonary vascular cells [31,32]. The accumulation of triglycerides, diacylglycerols, and ceramides is described in the RV of PAH patients. Increased CD36 transporter-mediated lipid uptake in PAH cardiomyocytes is present in patients with *BMPR2* mutations and in animal models [32,33]. However, patients with very severe PAH appear to have impaired fatty acid uptake in the RV, as suggested by single photon emission computed tomography (SPECT) using the fatty acid analog 123I-β-iodophenyl pentadecanoic acid. One hypothesis would be a limitation of the uptake of fatty acids by the failing RV [34,35]. Very interestingly, the enhanced absorption of fatty acids is not associated with the upregulation of FAO. Incomplete mitochondrial FAO is suggested by the elevation of lipid metabolites such as carnitine and acylcarnitine in the plasma of PAH patients [36].

As detailed above, in PAH, HIF1-α activation leads to the upregulation of glycolytic genes. This reprogramming decreases FAO, which is one of the mechanisms of lipid accumulation in cardiomyocytes and RV remodeling and altered function [37,38]. It was also demonstrated in pulmonary arterial ECs where FAO decreased [39].

Two key enzymes involved in FAO are upregulated in PAH. The first enzyme is carnitine palmitoyltransferase 1 (CPT1), which is upregulated in the lungs of monocrotaline rats and the pulmonary arteries of PAH patients. The overexpression of CPT-1, which allows the transfer of fatty acids into mitochondria, promotes the proliferation of SMCs and ATP production [40]. The second enzyme is malonyl-CoA decarboxylase (MCD). This enzyme catalyzes the reaction of malonyl-CoA to acetyl-CoA. MCD inhibition leads to an increase in the level of its substrate, malonyl-CoA, which decreases the oxidation of fatty acids, thereby lifting the inhibition of PDH and thus promoting glycolysis. As will be discussed in more detail later in this manuscript, MCD represents a target of choice in metabolic dysfunctions in PAH [41].

Furthermore, targeting the Randle cycle with FAO inhibitors increases RV glucose oxidation and RV function in experimental RV hypertrophy [30].

### 2.4. Electron Transport Chain (ETC)

Some studies suggest ETC abnormalities in PAH. The ETC is located in the inner membrane of the mitochondrion and consists of a series of five complexes (Complexes I, II, III, IV, and V, the ATP synthase). The flow of electrons from Complex I to IV generates a gradient of protons in the inner membrane that are used by ATP synthase to produce ATP. The expression and/or activity of ETC complexes are modified in the pulmonary arterial ECs of PAH patients as well as in animal models [8]. The first studies on mitochondrial respiration in PAH were performed in avian models of PH and showed decreased respiratory coupling in heart muscle [42]. James et al. demonstrated that chronic inhibition of Complex III in rats by Antimycin A induced pulmonary vasoconstriction though a decrease in the expression of keys proteins involved in FAO, the TCA cycle, ETC, and amino acid metabolism, as well as proteolysis and mitophagy [43,44]. Thus, targeting ETC complexes could modulate overall mitochondrial metabolism. Furthermore, a study of mitochondrial respiration in pulmonary vascular ECs from PAH patients showed a decrease in mitochondrial respiration (state 3 and 4) with a decrease in complex IV activity compared to controls [8]. The coupling between O_2_ consumption and ATP production, the respiratory control index, and Complex III activity were not modified. In this study, a decrease in the number of mitochondria was found in ECs of PAH patients. However, this study involved a small number of patients.

In addition, several studies have focused on the activity of respiratory chain complexes in RV cardiomyocytes or skeletal muscles. An increase in the expression and activity of Complex II (subunit B) associated with an increase in ROS production was found in the cardiomyocytes of animal models of PAH (rats exposed to monocrotaline) [45]. Another study published by the Amsterdam team showed in an animal model an improvement in RV myocardial fibrosis and an increase in vascular (capillary) density associated with an increase in mitochondrial capacity reflected by an increase in Complex II and Complex IV activity in rats exposed to monocrotaline and treated for PAH with Bosentan and Sildenafil. In this article, the authors correlated Complex II activity with the maximal O_2_ demand of cardiomyocytes [46]. Concerning the skeletal muscles of PAH patients, a decrease in Complexes I and III has been described [43,47,48,49]. The disturbance in the functioning of the ETC complexes induces a disturbance in the production of ROS, which is one of the other mechanisms contributing to PAH pathogenesis.

Interestingly, regarding the extravascular pulmonary cells, platelets of PAH patients demonstrated increased respiratory reserve capacity linked to an increase in FAO and Complex II activity [50]. These results correlated with hemodynamic parameters but were not modified by specific PAH treatments. More recently, Sommer et al. showed no change in mitochondrial respiration in peripheral blood mononuclear cells (PBMCs) from PAH patients compared to controls [51]. However, in this study, mitochondrial respiration was negatively correlated with disease severity according to hemodynamic parameters. All of these interesting data are preliminary and need to be confirmed by further studies, but specific targeting of ETC complexes could be an interesting therapeutic option [52].

### 2.5. Mitochondrial Membrane Hyperpolarization

Hyperpolarization of the mitochondrial membrane is one of the mechanisms leading to cell survival and proliferation. Mitochondrial membrane polarization, witnessed by the proton gradient, is essential for mitochondrial respiration. Certain factors are involved such as the protagonists of the STAT3 pathway, nucleolar factor of activated T-cells (NFAT), and Pim-1. These two proteins are responsible for the activation of Bcl2 (which is also an inhibitor of Kv channels) and Bad. When the membrane is hyperpolarized, a hypoxia-like signal is induced even in the presence of O_2_ [53]. The regulation of ionic concentrations between the cytoplasm, the mitochondrial intermembrane space, and the mitochondrial matrix is very important, and altering intracellular calcium levels deregulate intramitochondrial concentrations and thus inhibit the functions of metabolic enzymes, reducing mitochondrial activity, and promoting the Warburg effect. These factors therefore have the potential to induce HIF-1 activation, via mitochondrial hyperpolarization, or by acting indirectly on the activation of glycolysis enzymes.

### 2.6. Mitochondrial Dynamics

Beyond the Warburg effect, recent work has proposed a concept that, in PAH, altered mitochondrial dynamics (i.e., increased division–named fission–and decreased fusion) would promote the proliferation/resistance to apoptosis phenotype in pulmonary arterial cells. Typically, mitochondrial homeostasis is mediated by the coordination of mitochondrial biogenesis, dynamics, and mitophagy. Indeed, mitochondria can move, fragment (also called fission) or fuse, and mitochondrial division is crucial to maintain the number and distribution of mitochondria in cells and to transfer gene products between mitochondria [54]. In PAH, dysregulated mitochondrial fragmentation and mass reduction result in metabolic reprogramming [55]. Increased cytoplasmic GTPase dynamin-related protein 1 (DRP1) in pulmonary arterial SMCs, PPAR-γ coactivator (PGC1 α), and HIF-1 are involved in this mechanism [56]. Numerous per-clinical studies have shown that the downregulation of DRP1 by inhibitors can reverse PAH [56,57,58]. Because DRP1 phosphorylation leads to fission, DRP1 activity could be modulated by a variety of kinases. The phosphorylation of serine sites 637 and 656 reduces DRP1 activity, whereas the phosphorylation of serines 616, 579 or 600 causes mitochondrial fission by enhancing DRP1 activity [59,60,61,62]. In view of these data, targeting mitochondrial dynamics by modulating DRP1 activity is a therapeutic option for PAH. Marsboom et al. demonstrated that the activation of HIF-1 α led to mitochondrial fission via the phosphorylation of DRP1 serine 616, and the DRP1 inhibitors, mitochondrial division inhibitor 1 (Mdivi-1) and siDRP1, prevented mitochondrial fragmentation and reduced the proliferation of SMCs [63]. In addition to inhibiting mitochondrial fission, Mdivi-1 also inhibits glycolysis in pulmonary arterial SMCs [56]. Interestingly, however, Mdivi-1 is not only a specific inhibitor of DRP1. Bordt et al. showed that DRP1 inhibits mitochondrial complex I-dependent O_2_ consumption and reverses ROS production at concentrations used to target mitochondrial division [64]. In addition, the decrease in the miR-34a-3p-MiD pathway induced by the upregulation of the dynamics of the mitochondrial proteins of 49 and 51 kDa (MiD49 and MiD51) in PAH SMCs increases DRP1-mediated fission and promotes vascular proliferation [65]. This pathway would be an interesting target for PAH treatment.

On the other hand, the restoration of mitochondrial fusion would be another potential therapeutic mechanism in PAH. The main proteins involved in mitochondrial fusion are optical atrophy 1 (OPA1) and the GTPases mitofusin-1 and 2 (MFN1/2), and their inhibition reduces mitochondrial fusion and increases mitochondrial fission. In PAH, the upregulation of MFN2 can reduce the proliferation of SMCs and increase the apoptosis of SMCs by increasing mitochondrial fusion and reducing mitochondrial fission [66,67].

Finally, mitophagy required to maintain quality control by removing damaged mitochondria appears to be modified. Liu et al. demonstrated that mitophagy is actively involved in hypoxic PH by regulating pulmonary arterial SMCs [68]. The overexpression of FUNDC1 is thought to be one of the incriminated pathways by activating the ROS-HIF1α pathway and may become a novel therapeutic target for PAH.

### 2.7. Other Metabolic Pathways Outside of the Mitochondria: The Pentose Phosphate Pathway (PPP)

Apart from the Warburg effect, increased PPP has been observed in PAH patients and animal models and is thought to be involved in the development of the disease [69,70]. PPP is a metabolic pathway parallel to glycolysis and takes place in the cytosol of the cell. The two most important products from this process are ribose-5-phophate (R5P) a precursor of nucleotide synthesis, and nicotinamide adenine dinucleotide phosphate (NADPH), which is used in redox homeostasis within the cells. In PAH, an overproduction of NADPH has been demonstrated in the pulmonary microvascular ECs and SMCs [71,72]. In addition, the activity of glucose-6-phosphate dehydrogenase (G6PD), the primary rate-limiting enzyme of PPP, is enhanced in vitro or in the lung tissues of animal models of PAH related to a high proliferative state [69]. These data suggest that a decrease or deficiency of G6PD may protect against the development of PAH. However, the role of G6PD is multifactorial: it is involved in metabolism, oxidative stress, and red blood cell fragility [73]. G6PD deficiency reduces NO synthesis through its action on NADPH production or induces the activation of the p38 pathway, which could worsen PAH [74]. The benefit of targeting this pathway is therefore uncertain.

### 2.8. Deficiency of Iron–Sulfur (Fe–S) Biogenesis

Clinical links between PH and metabolic diseases have been suspected, leading to the classification of PH associated with metabolism syndromes in WHO group 5 [1]. Among the key components of mitochondrial metabolism in pulmonary ECs are Fe–S clusters, the alteration of which might be involved in the pathophysiology of PH [75]. Fe–S clusters are important cofactors of Complexes I, II, and III and are responsible for ETC dysfunction when altered. In humans, genetic mutations in the iron–sulfur cluster assembly protein or the BolA family member 3 or genetic mutations in iron–sulfur cluster scaffold protein (NFU1) genes, which are responsible for Fe–S cluster biogenesis and insertion into the ETC, induce exercise intolerance, myopathy, and death in some cases [75,76]. Interestingly, the development of PAH has also been described in some patients with these mutations [77,78]. These data are reinforced by preclinical models [75,79,80]. In addition, the deficiency of frataxin, a Fe–S biogenesis protein, generated the senescence of the pulmonary endothelium [81]. Furthermore, one of the mechanisms of hypoxia-inducible microRNA-210 (mir-210) in the physiopathology of PAH is thought to be the repression of the assembly of Fe–S clusters [82,83]. Very interesting, the genetic modification of NFU1 induced the decreased activity of Complex II and PDH [76]. The homozygous NFU1^G206C^ rat is the first animal model of PAH driven by a Fe–S biogenesis gene mutation. It has provided insight into the link between the Fe–S system and mitochondrial metabolic abnormalities in PAH. These data emphasize the interest of restoring the Fe–S system in PAH, and further studies are needed to better understand the intrinsic mechanisms related to mitochondrial dysfunction.

## 3. Current Therapeutic Targets of Mitochondrial Dysfunction in PAH

Based on these mechanisms, we can easily understand that therapies targeting mitochondrial metabolism are very promising in PAH. The promotion of mitochondrial OXPHOS, the restoration of mitochondrial imbalance, the inhibition of mitochondrial fission, and autophagy are all interesting therapeutic targets.

As described above, reducing glycolysis and restoring OXPHOS metabolism may be an effective therapeutic strategy. Through these mechanisms, dichloroacetate (DCA), a mitochondrial PDK inhibitor, can reverse the Warburg effect in pulmonary arterial SMCs and RV cardiomyocytes and lead to PAH improvement [84,85,86,87]. DCA is a competitor of pyruvate-PDK binding. It was first demonstrated in preclinical models. In addition, Li et al. suggested a complementary effect of DCA and atorvastatin on mitochondrial oxidative stress and the proliferation of pulmonary arterial SMCs through p38 activation [87]. The first clinical trial using DCA showed hemodynamic improvement with a significant reduction in mean PAP and PVR in patients with genetic susceptibility to PAH, confirming the interest of PDK as a therapeutic target [85]. However, some patients did not respond to DCA, and these patients had functional variants of SIRT3 and UCP2 that predict reduced protein activity, suggesting that PDH inhibition in these patients was less PDK dependent. In addition, DCA may have an effect on activated immune cells in PAH, but further studies are needed to explore this pathway [88]. Furthermore, the chronic administration of DCA is generally well tolerated, as described in children with congenital causes of lactic acidosis [89]. Further clinical studies are needed, and it would be interesting to combine DCA with a tyrosine kinase inhibitor, as tyrosine kinases can inhibit both PDH and active PDK [90].

Other potential therapeutic targets reducing glycolysis in animal models may be of interest, such as the glycolytic enzyme α-enolase inhibitor, the glycolytic regulator 6-phosphofructo-2-kinase/fructose-2, 6-bisphosphatase inhibitor, the 3-bromopyruvate (3-BrPA), a selective hexokinase 2 inhibitor, or the suppression of enolase 1 using AP-III-a4 (ENOblock) [91,92,93,94]. Inhibitors of nuclear factor of activated T-cells (NFAT), such as cyclosporine, are also attractive treatments in PAH because of their potential to regulate glycolysis. The effect of cyclosporine is also interesting because of its ability to inhibit HIF-1 transcriptional activity in the lungs [95]. However, these data are limited to preclinical models [96]. In addition, carvedilol, a beta-blocker, also decreases the rate of RV glycolytic activity as measured by FDG uptake [97].

By targeting HIF-1α, mammalian target of rapamycin (mTOR) inhibitors such as rapamycin or everolimus act on glycolysis in addition to their action on pulmonary vascular proliferation [98,99,100].

The reactivation of PDH can be achieved directly by the inhibition of PDK (using DCA) or indirectly by the activation of the Randle cycle using FAO inhibitors. Trimetazidine and ranolazine are two long-chain FAO inhibitors that improve RV function and treadmill walking distance in animal models [101,102]. The safety and efficacy of ranolazine were demonstrated in a pilot study of 11 PAH patients. In this study, it resulted in clinical and echocardiographic improvements, but no improvement was described in terms of hemodynamics [103]. These results were reinforced by a randomized controlled trial of 22 patients in which cardiovascular magnetic resonance imaging showed an increase in the RV ejection fraction [104]. Similarly, trimetazidine is well tolerated and appears to have a similar benefit in terms of outcome on RV function and functional and exercise capacity in PAH patients [105].

The inhibition of glutaminolysis is another attractive pathway to restore glucose oxidation. In an animal model, 6-Diazo-5-oxo-L-norleucine, a glutamine antagonist, improved RV function, reduced RV hypertrophy, and improved cardiac output [26]. Similarly, 2-hydroxyben-zylamine improved cardiac output in a *BMPR2*-mutant PH mouse model [25]. In humans, CB-839, an oral glutaminase inhibitor, has been studied in a phase 2 cancer trial (Clinical Trial NCT 02771626) and could therefore be repurposed for human PAH studies, especially given the results obtained by Bertero et al. in animal models [27]. Further, synergy studies of inhaled CB-839 associated with verteporfin have shown benefits in rat models of PAH [106].

In addition, the maximization of the ability of the TCA cycle to process the acetyl-CoA produced may be a complementary therapeutic approach that still needs to be explored. The inhibition of the pyruvate carboxylase anaplerosis reaction by phenylacetic acid is a potential therapeutic intervention demonstrated in sugen/hypoxic PAH rats but requires further investigation [107].

Beyond targeting mitochondrial metabolic abnormalities in PAH, therapeutics targeting mitochondrial fission are also promising in animal models. Therapies targeting DRP1 and its binding partners, miD49 and miD51, are under investigation [58]. For example, the inhibition of DRP-1 activity by Mdivi or SiDRP-1 reduces proliferation and induces cell cycle arrest [63]. Abu-hanna et al. suggested that treprostinil promotes mitochondrial fusion and elongation though the activation of PKA and the phosphorylation of DRP1 on the inhibitory residue serine 637 [108]. In addition, the upregulation of MFN2 or the activation of NRF-1 and heme oxygenase-1 is promising in pre-clinical studies [109].

Although not detailed in this review, targeting ROS induced by mitochondrial dysfunction has also been studied in PAH. Although results are promising in animal models, human studies are less convincing.

Last but not least, a growing number of studies suggest interest in mitochondrial transplantation to rescue mitochondrial dysfunction. In experimental models of PH, it confers beneficial effects on RV function, pulmonary artery remodeling, and vasoconstriction [110,111,112], but the long-term outcome with this treatment remains to be defined [113].

**Figure 2:**
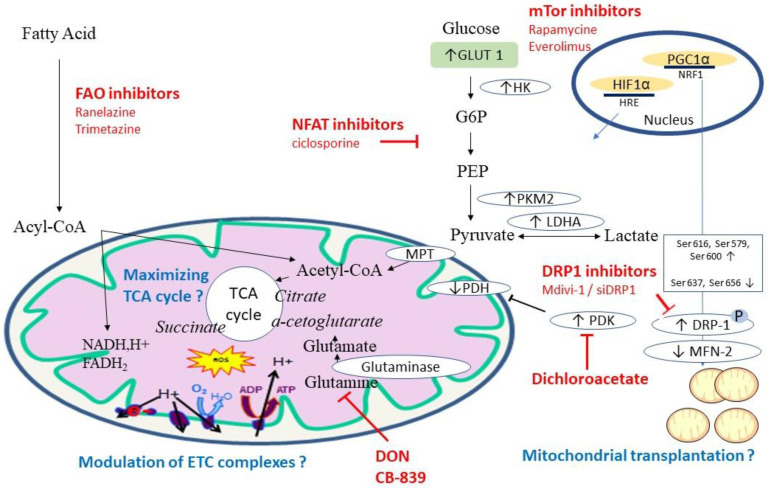
Current therapeutic options targeting mitochondrial dysfunction in PAH. Acyl-CoA: acyl-coenzyme A; DON: 6-Diazo-5-oxo-L-norleucine; DRP-1: dynamin-related protein 1; FAO: fatty acid oxidation; GLUT 1: glucose transporter 1; G6P: glucose-6-phosphate; HIF: hypoxia inducible factor; HK: hexokinase; HRE: hypoxia response element; LDHA: lactate dehydrogenase; Mdivi-1: mitochondrial division inhibitor 1; MFN-2: mitofusin-1; MPT: mitochondrial pyruvate transporter; mTOr: mammalian target of rapamycin; NFAT: Nuclear factor of activated T-cells; PDH: pyruvate dehydrogenase, PDK: pyruvate dehydrogenase kinase; PEP: phosphoenolpyruvate; PGC 1: Peroxisome proliferator-activated receptor-gamma coactivator; PKM2: pyruvate kinase isoform M2; TCA cycle: tricarboxylic acid cycle.

Current therapeutic options targeting mitochondrial dysfunction in PAH are summarized in Figure 2.

## 4. Conclusions

In conclusion, mitochondrial metabolic abnormalities are one of the mechanisms involved in the pathophysiology of PAH. This review highlights the diversity of metabolic pathways involved in PAH, including changes in glycolysis and increased glutaminolysis and FAO that are mediated by several enzyme systems and key transcription factors with HIF-1 α foremost. Targeting mitochondrial metabolism abnormalities in PAH seems to be a promising therapeutic strategy and could provide future advances in the treatment of the disease. Combined treatments targeting mitochondrial metabolism abnormalities seem to be complementary with the current therapies used in PAH given the different mechanisms targeted. In addition, given the positive results of the phase 3 trial of sotatercept in PAH, targeting the transforming growth factor beta superfamily, the modulation of pulmonary vascular remodeling is a novel target of interest [114]. However, currently tested therapies targeting mitochondrial metabolic dysfunction yield heterogeneous responses in PAH. This suggests that precision medicine in PAH should be promoted to select patients who would be most likely to benefit from these treatments.

## Data Availability

Data used for this review are published and therefore available as stated in the publications.
